# PANADA: Protein Association Network Annotation, Determination and Analysis

**DOI:** 10.1371/journal.pone.0078383

**Published:** 2013-11-12

**Authors:** Alberto J. M. Martin, Ian Walsh, Tomás Di Domenico, Ivan Mičetić, Silvio C. E. Tosatto

**Affiliations:** 1 Department of Biology, University of Padova, Padova, Italy; University of Copenhagen, Denmark

## Abstract

Increasingly large numbers of proteins require methods for functional annotation. This is typically based on pairwise inference from the homology of either protein sequence or structure. Recently, similarity networks have been presented to leverage both the ability to visualize relationships between proteins and assess the transferability of functional inference. Here we present PANADA, a novel toolkit for the visualization and analysis of protein similarity networks in Cytoscape. Networks can be constructed based on pairwise sequence or structural alignments either on a set of proteins or, alternatively, by database search from a single sequence. The Panada web server, executable for download and examples and extensive help files are available at URL: http://protein.bio.unipd.it/panada/.

## Introduction

The main protein sequence databases contain tens of millions of entries with many more sequences becoming continuously available due to the numerous genome sequencing efforts [1]. Currently, most known proteins lack any functional annotation [2] and very little is known about the vast majority. There are many ongoing projects trying to reduce the gap between known proteins and their functional annotation either computationally [3] or experimentally [4]. Recently there has also been the first Critical Assessment of Function Annotation (CAFA) experiment to assess the performance of function prediction methods [5]. Most computational approaches rely on pairwise similarity to known proteins to suggest functional annotations derived by homology to annotated database entries [6] [7]. Current methods still lack tools for the visualization of their results, in order to aid in their interpretation, analysis and to aid experts with curation. Precomputed pairwise comparisons with functional and structural annotations are available for instance in SIMAP [4], but one must build a similarity network by hand from this database. The Phytoscape framework [8] is available to build similarity networks, but it must be installed locally and offers a limited way to simplify large networks to be used in Cytoscape.

Protein sequence and structure similarity networks are bi-dimensional graphs where proteins are nodes with edges between them representing the pairwise similarity between the nodes they connect [9]. Such networks are increasingly being used for functional and structural protein annotation [10] [11]. They have also been used to detect errors in function annotation [12] and to study the evolution of multi-domain proteins [13]. Similarity networks complement phylogenetic trees and multiple sequence alignments, two more traditional approaches generally used to study and infer information derived from comparisons of protein sequences. The advantage of similarity networks is to leverage the human visual analytic skills to identify interesting patterns, e.g. of protein function or phylogenetic distribution, among a large protein set.

Here we describe PANADA, an automatic toolkit to visualize and study sequence and structure similarities between proteins to infer function by homology to other known proteins for use with the Cytoscape platform [14].

## Implementation

**Figure 1:**
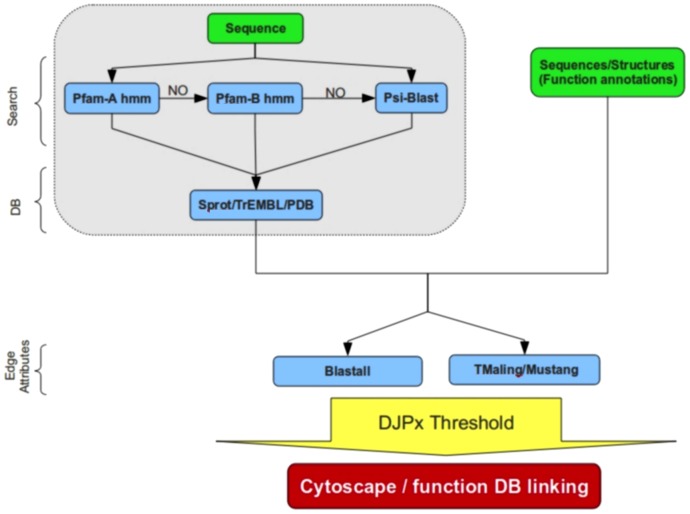
Flow chart of PANADA. An overview of alternative steps performed by PANADA is shown. Depending on the input, a set of proteins, sequences or structures, or a single sequence may be submitted. In the latter case PANADA uses Pfam and/or BLAST to find homologs. Functional annotations may be provided by the user if a set of sequences or structures is uploaded. In all cases the network is generated for a given similarity threshold and with a maximum number of edges per node (DJPx algorithm). The output can then be used in Cytoscape for visual analysis.

PANADA is available as both a web server and a Linux executable for download. The toolkit has been designed to be flexible, allowing the user to consider either protein sequences or structures. In similarity networks, nodes are protein sequences or structures. Edges represent associations between nodes, with a weight for the degree of similarity between nodes. PANADA operates either with input from an entire group or a single protein. Analysis of a group of sequences or structures is used to establish relationships among them. When a single protein is provided, the server first performs a search for close sequences or structures in publicly available databases. This can be especially useful to suggest functional annotations of uncharacterized proteins or to study relationships among different proteins belonging to the same family. Either way, proteins in the set are compared to each other in a pairwise manner. The overall workflow of PANADA is shown in Fig. 1.

Sequence similarity in PANADA is computed using BLASTALL [15], reporting pairwise local alignments measuring the percentage of sequence identity, E-value, bit score and alignment length. When generating structural similarity networks, PANADA compares protein structures using either MUSTANG [16] or TMalign [17]. TMalign computes root mean square distance (RMSD) or its scaled version TMscore, after a residue-to-residue alignment based on structural similarity using dynamic programming between two Cα traces. MUSTANG computes RMSD based solely on structural correspondence after Cα trace superimposition. TMscore, with values ranging between [0,…,1], is more sensitive to the topology of the protein structures being compared and less affected by local variations than RMSD. In general, TMscores below 0.17 mean the two compared proteins are structurally unrelated while proteins with scores above 0.5 share the same overall topology [18]. Due to the asymmetry of the comparison methods, each protein pair is compared in both directions, i.e. A vs. B and B vs. A, and the better value used.

PANADA can find related sequences from only a single input protein using Pfam protein family HMMs [16] and PSI-BLAST [15]. PDB [19] and the SwissProt and TrEMBL sections of UniProt [1] can be searched. In the initial step when using Pfam HMMs, a search is performed to identify to which Pfam family or families the query sequence belongs to. The search is first performed against Pfam-A, the manually curated set of protein families, and only when no significant match is found extended to Pfam-B. The identified Pfam HMMs are then used to search against the selected protein database for sequences containing the same functional region. PSI-BLAST is used only when the user desires to perform the initial database search using it or when there are no significant matches in Pfam. Proteins identified using PSI-BLAST could share only short stretches of local similarity or be biased due to the contents of protein databases [20]. PSI-BLAST parameters used in PANADA ensure that short regions are disregarded when query sequence and found hits share only one domain but the rest of the sequences are very different (i.e. belong to different domains). Due to the nature of the software used, multidomain proteins may however be problematic and require the user's judgment. Once the similarities between different proteins are computed, a selected measure is used to build the network by normalizing the similarity values in the range [0,..,1] (see online documentation). The highest value represents the shortest distance between the nodes in the network (greatest homology), and 0 the highest distance (lowest similarity). As PANADA produces multiple edges, the choice of measure is left to the user. This can be easily achieved by removing unwanted edges in Cytoscape through filtering.

Additionally, PANADA fetches GO [3] functional annotations for the compared proteins whenever available, i.e. for proteins with UniProt and PDB identifiers, with three different confidence levels. The user may select only experimental annotations, those considered reliable or everything. Reliable annotations include those inferred from electronic annotation (IEA) [3]. GO annotations of a node may be transferred to its neighbors without annotations, as the network explicitly represents similarity between proteins (property transfer by homology). GO terms can also be used to validate the annotations for single proteins or for all nodes in the network. If the same or related GO terms are present, these are more likely to be real. PANADA allows to color nodes according to their respective protein annotations in each of the three GO ontologies (molecular function, biological process and cellular component). Annotations for each protein are associated to their respective GO Slim and the most common GO Slim term for each node is given a hexadecimal ASCII color code that can be used to color the nodes in Cytoscape.

Since fully connected networks do not provide more information than standard pairwise comparison methods, e.g. BLAST search, removing edges in similarity networks increases the information content and enhances their interpretability [21]. PANADA implements two algorithms to reduce the number of connections present in a network. Edges are filtered either by leaving only edges representing high similarity (above a fixed threshold) or keeping the top X weighted edges for each node. The protocol used to keep the X top edges is a very simple modification of Prim's algorithm (DJPx). Prim's algorithm demonstrates that a minimum spanning tree (MSP) can be constructed on a graph (or network) by iteratively growing a tree from the minimum weight (i.e. highest similarity) edges connecting nodes not already attached to the MSP [22]. The DJPx algorithm used in PANADA generalizes Prim's algorithm by considering the top X edges instead of just one edge. Briefly, a single similarity measure is chosen to rank all normalized edges for all nodes from highest to lowest similarity. Starting from the highest similarity edge in the list, an edge is kept only if the nodes it connects do not yet have X edges. The selection is repeated until all nodes have X edges assigned and all remaining unassigned edges are removed. This ensures that the most relevant edges are kept and only low-quality ones are pruned. The two mechanisms to remove edges in the network, threshold and DJPx, can be used separately or combined. When both are combined in the final network, only the top X edges for each protein are kept while ensuring that they represent meaningful associations. When BLASTALL or TMalign are used to generate pairwise comparisons, connections between nodes are also removed if alignment coverage is lower than a predefined threshold.

The PANADA server produces a global output page with links to the downloadable output and Cytoscape network files as well as GO annotations and other relevant statistics. The output page includes normalized distance of direct neighbors to the query protein when using the automatic search option or all found GO terms for each protein when using a selected set of proteins. In both cases, the occurrence of each GO term belonging to proteins in the network is also shown.

## Usage Examples

**Figure 2:**
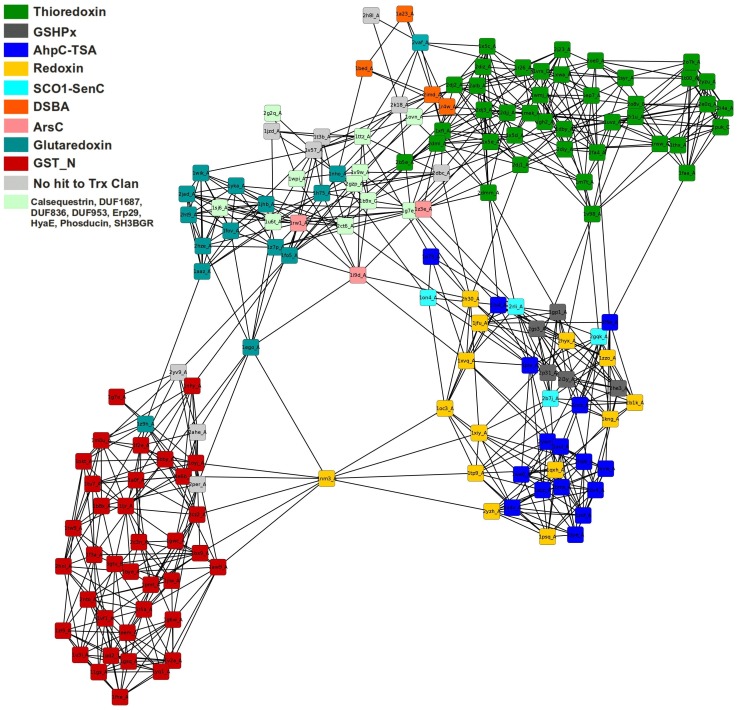
Protein similarity network of Thioredoxin-like structures. The PDB codes of structures from a previous publication [23] are used in PANADA to derive a network representation coloured by functional class. The organic layout was generated in Cytoscape with PANADA default parameters. The correspondence between colour codes and functional groups is shown in the upper left part. Notice how structures with the same functional class form tightly packed cluster separated from each other by a few connecting structures.

Figure 2 shows the results of a PANADA search of Thioredoxin fold class structures. The dataset contains the same structures as those used in a previous publication [23], with 159 protein chains at less than 60% sequence identity. The network was generated using MUSTANG to compare the 3D structures with default parameters. The results clearly separate the proteins into three main clusters representing the three main biological processes in which Thioredoxin fold class proteins are involved, showing how the overall structure of a protein chain relates to its catalytic function. This approach can be used to assign functional annotations inferred by homology of any query sequence and to determine possible misannotations and uncertainties within biologically related sets of proteins. For selected proteins of known structure, the PANADA analysis may be further combined with a residue interaction network analysis using RING [24] to determine the key structural components. Multiple sequence alignments will also provide complementary information about the proteins in the network and help to identify conserved residues that are likely to be related to protein function.

**Figure 3:**
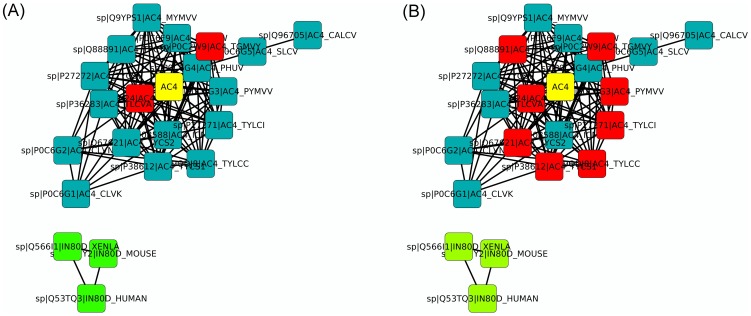
PANADA results of a sequence with no functional annotation. Starting from the viral protein AC4 (UniProt accession number P0DJX3), the constructed network is shown in Cytoscape with organic layout. (A) is colored by biological process with red for “virus-host interaction” (IEA) and green for “DNA recombination; DNA repair; regulation of transcription, DNA-dependent” (IEA). (B) is colored by cellular component with red for “host cell plasma membrane” (IEA) and green for “nucleus” (IEA). In both cases, the yellow color is used for the query protein AC4.

To demonstrate the use for single proteins with unknown function, Figure 3 shows the PANADA network for protein AC4 from Bean golden yellow mosaic virus (UniProt accession number P0DJX3), generated with by default parameters in the automatic search (SwissProt database and full GO annotation). This protein's existence was inferred by homology and although the genome is published [25], it was added to SwissProt on May 1, 2013. The two parts of Fig. 3 show the same network using the Cytoscape organic layout and only edges representing sequence identity. In the network, there are 22 different proteins. Eleven nodes have Cellular Component annotations, three “nucleus” (IEA) and eight “host cell plasma membrane” (IEA). Figure 3a shows the network colored according to the nodes Cellular Component annotations. According to the network, it is possible to infer AC4 cellular location GO terms to be “host cell plasma membrane” since the other annotations are in an unconnected cluster from the query protein. The same happens with the Biological Process annotations, see Figure 3b. Five proteins have Biological Process GO terms of two types, three with “DNA recombination; DNA repair; regulation of transcription, DNA-dependent; transcription, DNA-dependent” (all IEA); and two with “virus-host interaction” (IEA). For the same reasons as for Cellular Component one can infer AC4 terms as those proteins in the same subnet are likely to perform the same function.

**Figure 4:**
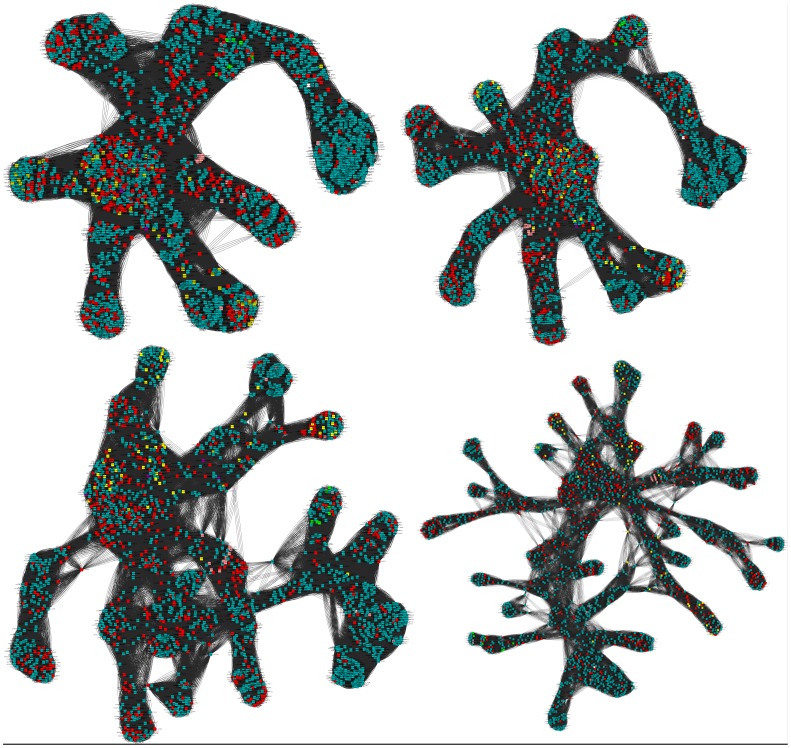
Variation of a protein similarity network as a function of DJPx threshold. An automatic search for E. coli protein YebC (PDB code 1KON) represented with Cytoscape organic layout and different maximum number of top edges per node (DJPx). Related proteins are found in UniProt database using the Pfam-A family PF01709. Edges are shown for pairwise sequence identity greater than 40% and alignment coverage at least 50%. From left to right and top to bottom, the networks shown the top 100 edges per node, 75, 50 and 25. In all cases, nodes sharing the Biological Process GO terms electronically assigned (IEA) to the query protein are colored in red.

To further explore the effects of parameters on using PANADA, we created several sequence networks using the automatic search for *E. coli* protein YebC (PDB code 1KON). This protein belongs to Pfam-A family PF01709 and until recently lacked GO terms. Currently it has the following IEA GO terms: Biological Process “regulation of transcription, DNA-dependent”, Molecular Function “DNA binding” and Cellular Component “cytoplasm”. Figure 4 shows networks created using alignment coverage of at least 50% and a maximum number of top edges per node (DJPx threshold) of 100, 75, 50 and 25. All other parameters were at default values. The networks contain 2,088 different proteins, of which 571 have at least one GO term associated. 413 proteins have exactly the same annotations as YebC, 71 share the same Molecular Function annotation as YebC, and 89 have several different Molecular Function terms. As can be seen in Figure 4, edge reduction by changing the maximun number of nodes simplifies the network. It is interesting to note how the network decomposes into local sub-clusters with decreasing threshold values. Since the closest YebC neighbors in the figure share the same GO terms, they confirm the electronically inferred annotations assigned to YebC.

## Conclusions

PANADA is a new online toolkit that generates protein similarity networks to be used with Cytoscape. PANADA allows the user to either automatically search similar sequences or to generate a network with a set of selected proteins. The similarity networks can be used for the visual analysis of similarity relationships among sequences or to asses functional annotation inferred from homology. PANADA complements other more traditional tools such as phylogenetic trees and multiple sequence alignments, making use of the user's visual skills to identify patterns that allow the inference of novel properties. The main advantages consist in the automatic search and annotation of proteins with GO terms from the database and the ability to choose two different approaches to prune the network topology. This produces networks that only contain edges for those pairwise comparisons that represent the highest similarities above a given threshold. Different utilities in Cytoscape, such as filters and the NetworkAnalyzer tools, add to the usefulness of PANADA providing the means for interpretation and analysis of similarity networks. PANADA automatically produces coloring based on the GO annotations of the proteins in the similarity network. Users can also define their own coloring scheme or their own annotations for each protein present in a network adding versatility to this toolkit. We anticipate PANADA to be of use for the visual analysis of protein function through similarity networks.
